# Supplementary material for: All-plasmonic sub-terahertz wireless communication link
Source: Nat Commun. 2025 Nov 13;16:9988. doi: 10.1038/s41467-025-64926-6 (PMC12615664; doi:10.1038/s41467-025-64926-6)
Supplement: Supplementary file 1 — Supplementary Information [file 41467_2025_64926_MOESM1_ESM.pdf]

Supplementary Materials for

Plasmonic-to-Plasmonic sub-Terahertz Wireless Communication Bridge

Tobias Blatter[1]*†, Stefan M. Koepfli[1]*†, Laurenz Kulmer[1], Amane Zuerrer[1], Samuel Hess[1], Yannik Horst[1], Marcel Destraz[2], Daniel Rieben[1], Michael Baumann[1], Jasmin Smajic[1], Yuriy Fedoryshyn[1] and Juerg Leuthold[1]*

1.ETH Zurich, Institute of Electromagnetic Fields (IEF), 8092 Zurich, Switzerland
2.Polariton Technologies AG, 8134 Adliswil, Switzerland

**corresponding email:* tobias.blatter@ief.ee.ethz.ch, stefan.koepfli@ief.ee.ethz.ch, juerg.leuthold@ief.ee.ethz.ch

The PDF file includes:

Supplementary Figures 1-2

Supplementary Table 1

Supplementary Note 1

Supplementary References 1-33

Supplementary Figure 1: Gate Dependent RF Response

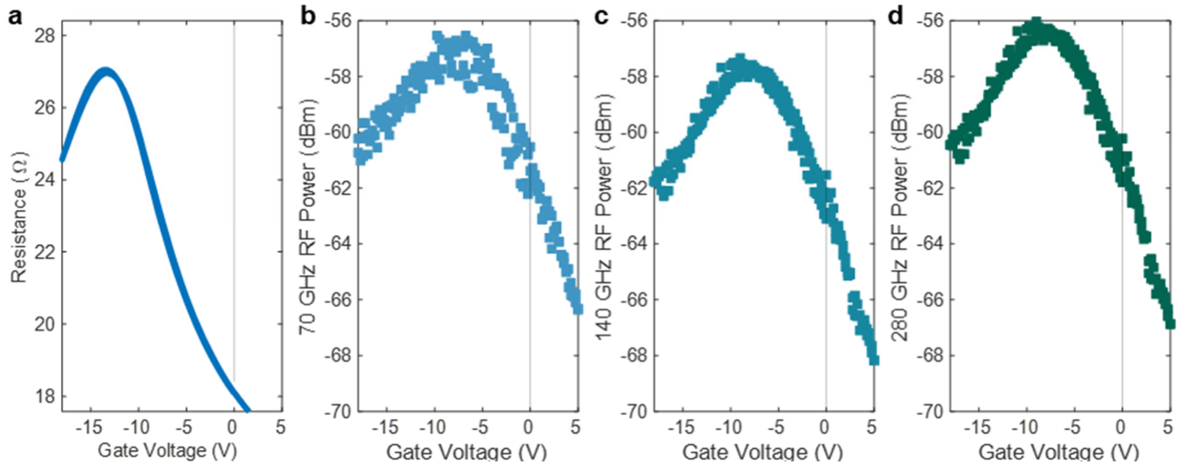

Figure S1: RF power dependence on gate voltage.
a) As reference, device resistance as function of gate voltage (main text Figure 2d). The measured RF power as function of gate voltage at b) 70 GHz, c) 140 GHz and d) 280 GHz.

Supplementary Figure 2: Schematic of Graphene Photodetector Dimensions

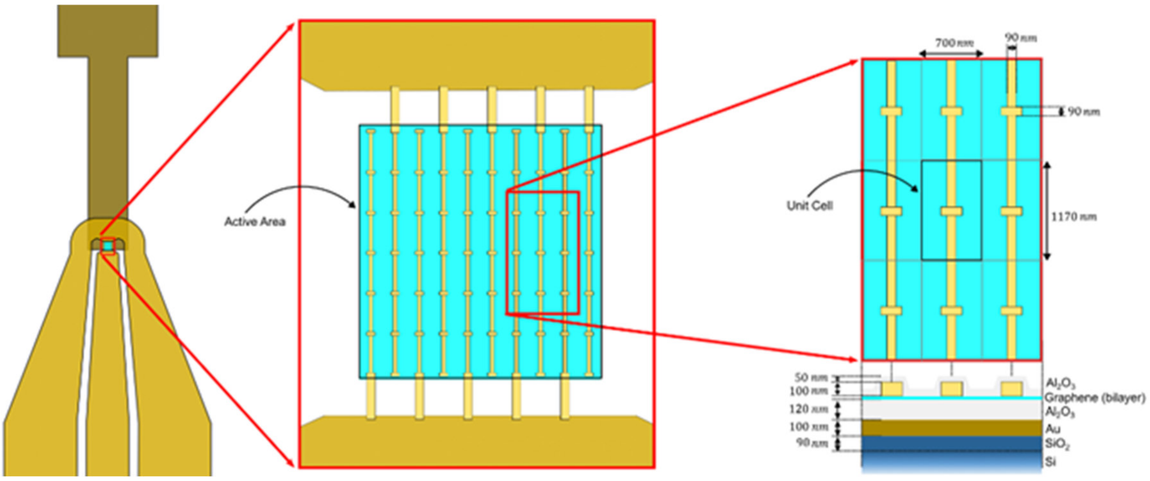

Figure S2: Schematic of the plasmonic graphene PD.
A visualization of the pad layout, the active area of the device and the metamaterial unit cell dimensions as described in the Methods section is shown. Additionally, a schematic of the layer stack is included.

Supplementary Table 1: Comparison of High-Speed Graphene Photodetectors

Ref.	Year	Bandwidth	Max. Output		Device Resistance	Det. Mech.	Responsivity	Bias Voltage	Gate Voltage
1	2009	>40 GHz	---	a	~2000 Ω	PV	0.5 mA/W	0 V	80 V
2	2010	16 GHz	~63 uA	b	140 Ω	PV	1.5 mA/W	0 V	-15 V
						-	6.1 mA/W	0.4 V	-15 V
3	2013	>20 GHz	~19.2 uA	c	---	PV	15.7 mA/W	0 V	No Gate
4	2013	18 GHz	~2.6 uA	-	---	PV	50 mA/W	0 V	No Gate
5	2014	3 GHz	~240 uA	-	~100 Ω	PC	57 mA/W	0.4 V	9 V
6	2014	41 GHz	~20 uA	-	187 Ω	PV	16 mA/W	0 V	No Gate
7	2015	42 GHz	~0.2 uA	b	98 Ω	PTE	78 mA/W	0 V	2.9 V
8	2016	65 GHz	~1 mV<br>~15 uA	a	254 Ω	PTE	3.5 V/W<br>35 mA/W	0 V	~6 V, ~ 6 V
						---	76 mA/W	0.3 V	~6 V, ~6V
9	2017	76 GHz	15.9 uA	a	130 Ω	BOL	1 mA/W	1 V	No Gate
10	2018	>18 GHz	1.5 mV<br>7.5 uA	x	200 Ω	PTE	4.7 V/W<br>48 mA/W	0 V	4 V, -2 V
						PC	170 mA/W	0.4 V	-8 V, 5 V
11	2018	> 50 GHz	3.7 uA	c	68 Ω	PC	1050 mA/W	0.02 V	-20 V
12	2018	>110 GHz	274 uA	b	100 Ω	BOL	400 mA/W	-0.6 V	No Gate
						PTE/PV	3 mA/W	0 V	No Gate
13	2018	38 GHz	~5.5 uA	-	300 Ω	---	0.57 mA/W	0 V	No Gate
14	2019	42 GHz	2.9 mV<br>~1.83 uA	x	~2000 Ω	PTE	12.2 V/W	0 V	-4 V,<br>-6 V
15	2019	>110 GHz	144 uA	d	100 Ω	PV/PC	360 mA/W	2.2 V	No Gate
16	2019	>70 GHz	15 uA	a	---	PC	300 mA/W	0.5 V	No Gate
17	2020	>67 GHz	4 mV<br>8 uA	a	~500 Ω	PTE	6 V/W<br>12 mA/W	0 V	3 V, -1 V
18	2020	>40 GHz	~87 uA	b	~150 Ω	BOL	396 mA/W	0.3 V to 1 V	0 to 2.8 V
19	2021	70 GHz	15.1 mV<br>~214 uA	b, x	~70 Ω	PTE	3.5 V/W	0 V	-1 V, -8 V
20	2021	70 GHz	208 uA	a	---	PC	104 mA/W	0.8 V	No Gate
21	2021	>67 GHz	---	a	172 Ω	PC	100 mA/W	0.3 V	No Gate
22	2021	12 GHz	38.6 mV<br>32.2 uA	b, x	~1200 Ω	PTE	90 V/W<br>75 mA/W	0 V	-0.5 V, -2.1 V
23	2022	78 GHz	233 uA	b	231 Ω	BOL	603.9 mA/W	1.9 V	No Gate
24	2023	30 GHz	0.21 uA	-	355 Ω	IPE/PTFE	0.33 mA/W	6 V	No Gate
25	2023	>500 GHz	45.8 uA	-	235 Ω	PV	1.57 mA/W	0 V	-2.5 V
26	2024	36 GHz	142 uA	b	150 Ω	PC	460 mA/W	0.5 V	No Gate
27	2024	420 GHz	18.5 mV<br>5.4 uA	b	5806 Ω	PTE	1.6 V/W<br>0.4 mA/W	0 V	0.5 V
28	2025	>14 GHz	17 uA	b	286 Ω	BOL	122 mA/W	2.6 V	No Gate
29	2025	155 GHz	---	a	255 Ω	PV/PTE/PC	68 mA/W	0.3 V	-38 V
**This work**	2025	>330 GHz	556 uA	-	22 Ω	PV	6 mA/W	0 V	-6.75 V

Table S1: Comparison of high-speed graphene photodetectors.
The table lists reported high-speed graphene photodetectors sorted by their publication year. Bandwidth values given with a ">" symbol indicate a setup limited measurement and not a device limitation. The output power have the following indications given in the column behind: a: no power sweep was performed, b: loss of linearity was observed, c: full saturation of the output was measured, x: the value was converted from photovoltage to photocurrent by using the device resistance.

Supplementary Note 1: Cost Discussions of High-Speed Photodetectors

For sub-THz transmission with signal generation based on opto-electronic converters, there are conventionally uni-travelling carrier photodiodes (UTC-PD) employed. The here proposed high-speed graphene photodetectors could offer a potentially cost-effective alternative with scalable fabrication processes.

The cost of high-speed photonic devices is essentially dominated by:
(1) Material cost
(2) Processing cost
(3) Packaging and co-integration

We outline below along these three points why plasmonic graphene photodetectors could be advantageous over UTC-PD.

(1) Material cost
- UTC PDs are commonly grown on InP wafers. Most epitaxy system rely on 2-inch or 3-inch wafers, where some systems are also available for 4-inch processing. The cost of the raw InP wafers is in the order of ~4 $/cm^2$. The epitaxy growth requires MOCVD/MBE systems that are expensive in procurement as well as in maintenance and in operation. Making a clear estimate on cost per run is difficult but is at least in the order of $/cm^2$.
- Graphene growth on copper foil with a CVD tool results in a cost of 0.019 $/cm^2$ as reported in[30]. This cost could even further be reduced by re-using the growth substrate after transfer[31]. Transfer can be done on almost arbitrary substrates. Considering a silicon wafer as in this work, the cost for the substrate is ~0.4 $/cm^2$.
The cost for the active material stack is thereby expected to be at least an order of magnitude lower than for III-V materials.

(2) Processing cost
Processing the active material to form functional devices is expected to be similar, as both require similar processing technologies. However, handling of Si wafers is typically much easier than handling InP wafers due to lower fragility, higher temperature tolerance, chemical robustness and low toxicity. Additionally, most available equipment is built around Si wafers which makes handling and automatization directly compatible.

(3) Packaging and co-integration
The steps for packaging are expected to have the same cost for both device technologies. However, due to the substrate independence of graphene it is possible to allow for direct co-integration of graphene with e.g. electronic circuits. An example demonstration is the direct integration of graphene with a CMOS camera read-out circuit[32]. The co-integration of plasmonic devices has also been demonstrated with high-speed electronics[33].

Supplementary References

1. Xia, F., Mueller, T., Lin, Y., Valdes-Garcia, A. & Avouris, P. Ultrafast graphene photodetector. *Nature Nanotech* **4**, 839–843 (2009).
2. Mueller, T., Xia, F. & Avouris, P. Graphene photodetectors for high-speed optical communications. *Nature Photon* **4**, 297–301 (2010).
3. Gan, X. *et al.* Chip-integrated ultrafast graphene photodetector with high responsivity. *Nature Photon* **7**, 883–887 (2013).
4. Pospischil, A. *et al.* CMOS-compatible graphene photodetector covering all optical communication bands. *Nature Photon* **7**, 892–896 (2013).
5. Youngblood, N., Anugrah, Y., Ma, R., Koester, S. J. & Li, M. Multifunctional Graphene Optical Modulator and Photodetector Integrated on Silicon Waveguides. *Nano Lett.* **14**, 2741–2746 (2014).
6. Schall, D. *et al.* 50 GBit/s Photodetectors Based on Wafer-Scale Graphene for Integrated Silicon Photonic Communication Systems. *ACS Photonics* **1**, 781–784 (2014).
7. Shiue, R.-J. *et al.* High-Responsivity Graphene–Boron Nitride Photodetector and Autocorrelator in a Silicon Photonic Integrated Circuit. *Nano Lett.* **15**, 7288–7293 (2015).
8. Schuler, S. *et al.* Controlled Generation of a p–n Junction in a Waveguide Integrated Graphene Photodetector. *Nano Lett.* **16**, 7107–7112 (2016).
9. Schall, D., Porschatis, C., Otto, M. & Neumaier, D. Graphene photodetectors with a bandwidth >76 GHz fabricated in a 6″ wafer process line. *J. Phys. D: Appl. Phys.* **50**, 124004 (2017).
10. Schuler, S. *et al.* Graphene Photodetector Integrated on a Photonic Crystal Defect Waveguide. *ACS Photonics* **5**, 4758–4763 (2018).
11. Cakmakyapan, S., Lu, P. K., Navabi, A. & Jarrahi, M. Gold-patched graphene nano-stripes for high-responsivity and ultrafast photodetection from the visible to infrared regime. *Light Sci Appl* **7**, 20 (2018).
12. Ma, P. *et al.* Plasmonically Enhanced Graphene Photodetector Featuring 100 Gbit/s Data Reception, High Responsivity, and Compact Size. *ACS Photonics* **6**, 154–161 (2019).

13. Gao, Y., Tsang, H. K. & Shu, C. A silicon nitride waveguide-integrated chemical vapor deposited graphene photodetector with 38 GHz bandwidth. *Nanoscale* **10**, 21851–21856 (2018).

14. Muench, J. E. *et al.* Waveguide-Integrated, Plasmonic Enhanced Graphene Photodetectors. *Nano Lett.* **19**, 7632–7644 (2019).

15. Ding, Y. *et al.* Ultra-compact integrated graphene plasmonic photodetector with bandwidth above 110 GHz. *Nanophotonics* **9**, 317–325 (2020).

16. Wang, Y. *et al.* 72 GBd graphene-on-plasmonic slot waveguide photodetector. in *45th European Conference on Optical Communication (ECOC 2019)* 1–3 (2019). doi:10.1049/cp.2019.0822.

17. Mišeikis, V. *et al.* Ultrafast, Zero-Bias, Graphene Photodetectors with Polymeric Gate Dielectric on Passive Photonic Waveguides. *ACS Nano* **14**, 11190–11204 (2020).

18. Guo, J. *et al.* High-performance silicon−graphene hybrid plasmonic waveguide photodetectors beyond 1.55 µm. *Light Sci Appl* **9**, 29 (2020).

19. Marconi, S. *et al.* Photo thermal effect graphene detector featuring 105 Gbit s−1 NRZ and 120 Gbit s−1 PAM4 direct detection. *Nat Commun* **12**, 806 (2021).

20. Wang, Y. *et al.* Ultra-Compact High-Speed Polarization Division Multiplexing Optical Receiving Chip Enabled by Graphene-on-Plasmonic Slot Waveguide Photodetectors. *Advanced Optical Materials* **9**, 2001215 (2021).

21. Wang, Y. *et al.* Ultrahigh-speed graphene-based optical coherent receiver. *Nat Commun* **12**, 5076 (2021).

22. Schuler, S. *et al.* High-responsivity graphene photodetectors integrated on silicon microring resonators. *Nat Commun* **12**, 3733 (2021).

23. Yan, S., Zuo, Y., Xiao, S., Oxenløwe, L. K. & Ding, Y. Graphene photodetector employing double slot structure with enhanced responsivity and large bandwidth. *OEA* **5**, 210159–10 (2022).

24. Guo, J. *et al.* High-Speed Graphene–Silicon–Graphene Waveguide PDs with High Photo-to-Dark-Current Ratio and Large Linear Dynamic Range. *Laser & Photonics Reviews* **17**, 2200555 (2023).

25. Koepfli, S. M. *et al.* Metamaterial graphene photodetector with bandwidth exceeding 500 gigahertz. *Science* **380**, 1169–1174 (2023).

26. Wu, Q. *et al.* Waveguide-integrated twisted bilayer graphene photodetectors. *Nat Commun* **15**, 3688 (2024).

27. Koepfli, S. M. *et al.* Controlling photothermoelectric directional photocurrents in graphene with over 400 GHz bandwidth. *Nat Commun* **15**, 7351 (2024).

28. Cai, H., Yang, C., Shen, L., Yu, Y. & Zhang, X. High-Efficiency and Polarization-Independent Waveguide-Integrated Graphene Plasmonic Photodetectors Operating at 2 µm. *ACS Photonics* **11**, 1565–1573 (2024).

29. Rieben, D. *et al.* Towards 200 GBaud Line Rates with Waveguide-Integrated Plasmonic Graphene Photodetectors. in *Optical Fiber Communication Conference (OFC) 2025 (2025), paper Th3E.4* Th3E.4 (Optica Publishing Group, 2025). doi:10.1364/OFC.2025.Th3E.4.

30. Goldsmith, B. R. *et al.* Digital Biosensing by Foundry-Fabricated Graphene Sensors. *Sci Rep* **9**, 434 (2019).

31. Gupta, P. *et al.* A facile process for soak-and-peel delamination of CVD graphene from substrates using water. *Sci Rep* **4**, 3882 (2014).

32. Goossens, S. *et al.* Broadband image sensor array based on graphene–CMOS integration. *Nature Photon* **11**, 366–371 (2017).

33. Koch, U. *et al.* A monolithic bipolar CMOS electronic–plasmonic high-speed transmitter. *Nat Electron* **3**, 338–345 (2020).
